# Influence of Genetic Selection for Growth and Broodstock Diet n-3 LC-PUFA Levels on Reproductive Performance of Gilthead Seabream, *Sparus aurata*

**DOI:** 10.3390/ani11020519

**Published:** 2021-02-17

**Authors:** Shajahan Ferosekhan, Serhat Turkmen, Cathaysa Pérez-García, Hanlin Xu, Ana Gómez, Nazeemashahul Shamna, Juan Manuel Afonso, Grethe Rosenlund, Ramón Fontanillas, Anselmo Gracia, Marisol Izquierdo, Sadasivam Kaushik

**Affiliations:** 1 IU-ECOAQUA, Universidad de Las Palmas de Gran Canaria, Taliarte, 35214 Telde, Spain; turkmen@uab.edu (S.T.); cathaysa.perez108@alu.ulpgc.es (C.P.-G.); hanlinxuulpgc@outlook.com (H.X.); juanmanuel.afonso@ulpgc.es (J.M.A.); anselmo.gracia@ulpgc.es (A.G.); marisol.izquierdo@ulpgc.es (M.I.); sachi.kaushik@ulpgc.es (S.K.); 2 ICAR-Central Institute of Freshwater Aquaculture, Bhubaneswar 751002, Odisha, India; 3 Department of Biology, University of Alabama at Birmingham, Birmingham, AL 35294, USA; 4 Institute of Aquaculture Torre de la Sal (IATS), CSIC, Ribera de Cabanes, 12595 Castellón, Spain; a.gomez@csic.es; 5 ICAR-Central Institute of Fisheries Education, Mumbai 400061, Maharashtra, India; shamna@cife.edu.in; 6 Skretting Aquaculture Research Centre, Sjohagen, 4016 Stavanger, Norway; Grethe.Rosenlund@skretting.com (G.R.); ramonfontanillas@gmail.com (R.F.)

**Keywords:** broodstock, fatty acid, genetic selection, spawning quality, sperm quality, steroid hormone

## Abstract

**Simple Summary:**

Gilthead seabream (GSB) broodstock were genetically selected based on their growth trait either high growth (HG) or low growth (LG) to evaluate the reproductive performance of these both traits under either fish oil (FO) or vegetable oil (VO) based diets feeding regime. The egg and larval quality were significantly improved by the broodstock selected for HG trait fed under FO based diet. This indicates that broodstock selected with HG trait has positive influence on the improving sperm, egg and larval quality in gilthead seabream.

**Abstract:**

Genetic selection in gilthead seabream (GSB), *Sparus aurata,* has been undertaken to improve the growth, feed efficiency, fillet quality, skeletal deformities and disease resistance, but no study is available to delineate the effect of genetic selection for growth trait on GSB reproductive performance under mass spawning condition. In this study, high growth (HG) or low growth (LG) GSB broodstock were selected to evaluate the sex steroid hormones, sperm, egg quality and reproductive performance under different feeding regime of commercial diet or experimental broodstock diet containing either fish oil (FO) or vegetable oil (VO) based diet. Under commercial diet feeding phase, broodstock selected for either high growth or low growth did not show any significant changes in the egg production per kg female whereas egg viability percentage was positively (*p* = 0.014) improved by the high growth trait broodstock group. The experimental diet feeding results revealed that both growth trait and dietary fatty acid composition influenced the reproductive performance of GSB broodstock. In the experimental diet feeding phase, we observed high growth trait GSB males produced a higher number of sperm cells (*p* < 0.001) and also showed a higher sperm motility (*p* = 0.048) percentage. The viable egg and larval production per spawn per kg female were significantly improved by the broodstock selected for high growth trait and fed with fish oil-based diet. This present study results signifies that gilthead seabream broodstock selected on growth trait could have positive role in improvement of sperm and egg quality to produce viable progeny.

## 1. Introduction

As one of the major species under aquaculture in the Mediterranean region, the gilthead seabream (GSB) (*Sparus aurata*) is subject to selective breeding programmes [1,2]. Selective breeding can be practiced following different methods, from the simplest mass selection to index selection and marker-assisted selection for production of offspring with desired traits. As mating system, all selection methods mainly use mass spawning in order to produce large quantities of the progeny required for selection process [2,3]. Conventional mass spawning has certain limitations for selection programs, since it may not be possible to identify the individual offspring’s parents. To overcome this issue, microsatellite markers are used as a powerful tool to reconstruct the genealogy among offspring and breeders [4].

Broodstock selection in fish has been based on somatic growth [5], feed efficiency [6], disease resistance [7], deformities [8,9], fillet yield [10,11,12] or fillet fatty acid composition [13,14]. Selective breeding programs in gilthead seabream have also addressed improved growth performance, disease resistance and carcass quality [15]. Assessment of parental contributions in fast- and slow-growing progenies of gilthead seabream has been also assessed using a multiplex PCR [16]. 

In Europe, 31 to 44% of gilthead seabream seed comes from breeder selection processes [2], mainly for growth performance and morphology as selected traits [17] and genetic improvement per generation of 5–29% [1,3]. Seabream broodstock selection by Best linear unbiased prediction (BLUP) methodology has been based on somatic growth and morphology in commercial hatcheries [5], as well as on growth, carcass, flesh and fish quality and disease resistance on a series of jointly projects from commercial and public research hatcheries, known as PROGENSA [15]. In those projects, a weak association was found between families selected for growth (low and high Estimated Breeding Values (EBV)) and the type of diet (fish or plant ingredients based diet), denoting that selection for faster growth is linked with different growth trajectories and a high diet flexibility and intestinal plasticity [18]. However, the possible impact of genetic selection for somatic growth on reproductive performance of gilthead seabream is not well documented. 

Gilthead seabream is a multi-batch spawner and egg quality relies very much on the continuous intake of nutrients to complete vitellogenesis during the whole spawning season. Therefore, adequate amounts of essential n-3 long-chain- polyunsaturated fatty acids (LC-PUFA) must be provided in broodstock diets for the proper gonadal and embryonic development in this species [19]. Given the limited availability of marine oils rich in LC-PUFA, recent work has also analysed the effects of diets containing alternative lipid sources such as microalgae [20,21] or krill oil [22,23], besides terrestrial vegetable oils (VO). It has been shown that partial replacement of fish oil by VO does not affect the spawning quality in gilthead seabream [24,25,26]. In all these previous studies, the GSB was selected based on the fatty acyl desaturase 2 gene (*fads2*) as a potential biomarker. In our previous study, Gilthead seabream broodstock selected for high *fads2* gene expression exhibited improved egg and larval quality, even when fed with a low fish oil diet [24,25,26]. Thus, high *fads2* broodstock fed relatively high amounts of linoleic (LA) and linolenic (ALA) acids showed an increased biosynthesis of n-3 LC-PUFA [26], and the subsequent nutritional programming improved the utilisation of low fish meal (FM) and fish oil (FO) diets by the progeny [25,27]. This nutritional programming effect of feeding broodstock with low FO diets persisted in the progeny even up to the 16 months old juveniles [28], and all the previous studies were based on the fatty acyl desaturase (*fads2*) as the potential biomarker to evaluate the reproductive performance of GSB. However, this present study was evaluated to ascertain the broodstock selection for growth trait (HG or LG) and fed with either FO or VO on the reproductive performance of GSB.

Therefore, the present study was initiated to analyse the effect of broodstock selected for growth (HG or LG) and dietary fatty acid source (FO or VO) on the reproductive performance of the gilthead seabream under mass spawning programme and the evaluation of HGFO broodstock performance over the LGVO broodstock. For that purpose, gilthead seabream broodstock selected for high (HG) or low (LG) growth were fed with two diets containing different lipid sources and based on fish oil (FO) or vegetable oil (rapeseed and linseed oil) along 3 months of the spawning season. The effect of the broodstock selection and the broodstock diet on the seabream reproductive performance, plasma sex hormones levels and egg biochemical and fatty acid composition were studied under a two-way ANOVA design.

## 2. Materials and Methods

### 2.1. Ethical Statement

The study was conducted according to the European Union Directive (2010/63/EU) on the protection of animals for scientific purposes at Aquaculture Research Group (GIA) of ECOAQUA Institute, University of Las Palmas de Gran Canaria (ULPGC), Canary Islands, Spain. All experimentation performed at the (ULPGC) was approved by the Bioethical Committee of the University of Las Palmas de Gran Canaria (REF: 007/2012 CEBA ULPGC).

### 2.2. Experimental Broodstock

The gilthead seabream broodstock used originated from third generation of selection under the PROGENSA (Spanish National Breeding Program) project [15]. Two broodstock groups expressing either high growth (HG), or low growth (LG), selected by BLUP methodology with VCE-v 6.0 software [29], were used for the assessment of reproductive performance in mass spawning. HG and LG trait broodstock were individually marked with PIT tags (EID Iberica SA-TROVAN, Madrid, Spain) and maintained separately for mass spawning in four tanks (10 m^3^) at the facilities of ECOAQUA institute (ULPGC, Canary Islands, Spain). All the tanks were supplied with seawater (37 g L^−1^ salinity, 17.8–19.0 °C) at a water exchange of 600% daily and maintained under natural photoperiod. The four broodstock groups were maintained separately for the whole reproductive season as HG (tanks 1 and 2) and LG (tanks 3 and 4), with an average biomass of 49 kg/tank and an average ratio female/male biomass range from 1.4 to 1.7 (Table 1). Male body weight and female weight and size were larger in HG than in LG broodstock (Table 1).

### 2.3. Phase I: Evaluation of Spawning Quality before Feeding the Experimental Diet

The selected males and females from LG and HG broodstock groups were assessed for the spawning quality. At the beginning of the spawning season, from 20 December 2018 to 23 January 2019, brood fish were fed with a commercial diet (Vitalis CAL, Skretting, Burgos, Spain) to ensure that there were no significant differences in the spawning quality among brood fish from the same selection group (HG or LG). Samples for analysis of sex steroid hormones and sperm quality were collected, and analysis procedure is described in below sections. Liver and gonad were excised and weighed for the determination of hepato-somatic index (HSI) and gonado-somatic index (GSI), respectively. Both these body indices were calculated as a percentage of organ to the whole-body weight of individual broodfish. For the evaluation of spawning quality, the spontaneously spawned eggs from each broodstock group were collected six times per week. Eggs were also collected at the end of the feeding period and kept at −80 °C until biochemical analysis. 

### 2.4. Phase II: Evaluation of Mass Spawning Quality after Feeding the Experimental Diets

The experimental broodstock feeds were formulated to be iso-nitrogenous and iso-lipidic with either fish oil (FO diet) or a mixture of vegetable oils (VO diet, containing rapeseed and linseed oil) as the lipid source and were produced by Skretting ARC, Stavanger, Norway (Table 2). Compared to the FO diet, the VO diet had higher levels of 18:2n-6 and 18:3n-3 fatty acids and reduced levels of saturated, monoenoic and n-3 LC-PUFA (20:5n-3; eicosapentaenoic acid, EPA and 22:6n-3; docosahexaenoic acid, DHA) (Table 3). The broodstock treatment groups were assigned as follows: HGFO, HGVO, LGFO and LGVO. Both the HG and LG brood fish groups were fed with one of the two diets at the rate of 1% body weight, twice a day (9:00 and 14:00 h), over a period of 3 months (24 January 2019 to 26 April 2019). Seawater temperature during broodstock spawning period was in the range of 18 to 22 °C (January-April 2019) and fish were kept under natural photoperiod. Egg collection for spawning quality and biochemical composition followed the same protocol described in the commercial diet feeding phase. Finally, after 3 months of feeding the two experimental diets, eggs were collected from all brood fish groups (HGFO, HGVO, LGFO and LGVO) and analysed for egg biochemical and fatty acid composition.

### 2.5. Plasma Sex Steroid Hormones

All the broodstock were fasted overnight and anesthetized with clove oil (10 ppm clove oil:methanol (50:50) in sea water) to collect blood samples. Blood was taken from the caudal vein using sterile syringes (Terumo Europe NV, Leuven, Belgium) and transferred to 3.0 mL K3-EDTA tubes (L.P. Italiana, Milan, Italy). Whole blood samples were centrifuged at 3000× *g* for 10 min at 4 °C, and plasma was separated and stored at −80 °C for sex steroid hormone analyses [26]. Plasma sex steroids were measured by enzyme immunoassays (EIA) as described for European sea bass for testosterone (T) [30], 11-ketotestosterone (11-KT) [31] (and 17β-estradiol (E2) [32]. Plasma steroids were extracted with methanol and supernatants were dried and reconstituted in EIA buffer (potassium phosphate 0.1 M, pH7.4 containing 0.01% sodium azide, 0.4 M NaCl, 0.001 M EDTA and 0.1% BSA). The assays were performed in 96-well plates coated with mouse anti-rabbit IgG monoclonal antibodies (Sigma-Aldrich, R-1008). Steroid standard curves (ranging from 0.0024 to 5.0 ng/mL for T; 0.0005–1.0 ng/mL for 11-KT and 0.039–80.0 ng/mL for E2; Sigma-Aldrich) or plasma samples were run in duplicate and added to the wells together with the corresponding. acetylcholinesterase (AChE) tracer: (T-AchE, 11-KT-AChE or E2-AChE; Cayman Chemical, Michigan, USA) and rabbit antiserum (anti-T, anti-11-KT or anti-E2), and incubated at 37 °C (E2) or 4 °C (T and 11-KT). Next, plates were rinsed, and colour development was performed by addition of Ellman reagent. Optical density was read at 405 nm using a microplate reader (Bio-Rad 3550). The sensitivities of the assays (80% of binding) were 0.025 ng/mL for T, 0.0049 ng/mL for 11-KT and 0.30 ng/mL for E2. The inter-assay coefficients of variation at 50% of binding were 0.42% with a 0.90 slope for T, 32.6% with a 0.84 slope for 11-KT and 2.05% with a 0.83 slope for E2. The intra-assay coefficients of variation were 2.93% (*n* = 4) for T, 5.65% (*n* = 6) for 11-KT and 0.83% (*n* = 4) for E2. Sex steroid hormone concentration values are presented as mean ± SD.

### 2.6. Sperm Quality Assessment

For sperm collection, fish were anesthetized as mentioned above, and sperm was collected from the blot dried genital pore after a gentle abdominal massage to induce spermiation and taking care to avoid contamination with water, faeces or urine. The collected sperm was stored on ice until transferred to a 4 °C refrigerator [26]. The sperm quality parameters that were evaluated included sperm concentration (number of spermatozoa/mL sperm, 10^9^ mL^−1^), sperm motility % (percentage of spermatozoa showing forward motility) and sperm motility duration (min). Sperm concentration was estimated after a 1000-fold dilution with sperm inactivation media using a Neubauer haematocytometer under 400× magnification. Sperm motility and motility duration were evaluated on a microscope slide (400× magnification) after mixing 1 µL of sperm with 50 μL of seawater [33,34].

### 2.7. Evaluation of Egg and Larval Quality

The spontaneously spawned eggs were collected and placed in 10 L containers provided with aeration for sample homogenization. There randomized 10 mL egg samples were taken and placed in a Bogorov chamber under the light microscope to calculate the total number of eggs (fecundity parameter) and percentages of fertilized, unfertilized and viable eggs. Egg viability was determined by observing the percentage of morphologically normal eggs after 1-day post fertilization (1 dpf) [35]. Then, the viable eggs were individually placed in 96-well microtiter plates in two replicates filled with filtered and UV sterilized seawater. Eggs were incubated in a controlled temperature incubator at 19–21 °C to estimate the percentage of hatching (2 dpf) and larval survival rates at 3 days post hatch (dph). From these values, all the spawning quality parameters were calculated such as, total numbers of fertilized eggs, viable, hatched and larvae produced per kg female [19].

### 2.8. Biochemical Analysis

After feeding either the commercial diet or the experimental conditioning diets, egg samples were collected from all the broodstock groups and stored at −80 °C for analysis of proximate and fatty acid composition. Moisture contents were obtained after drying the samples in an oven at 110 °C for 24 h and then for 1 h until constant weight. Ash content was determined after incineration at 600 °C for 16 h. Crude protein content was determined by measuring the N content (N × 6·25) through automated Kjeldahl analysis [36], and crude lipid extraction was carried out with chloroform: methanol [37]. Egg fatty acids methyl esters (FAMES) from total lipids were prepared by transmethylation method with 1% sulfuric acid in methanol, purified on NH_2_ silica (Sep-pak; Waters), and separated and quantified in a gas chromatograph (GC14A; Shimadzu, Kyoto, Japan) equipped with a flame ionization detector and a Carbowax 20 M (30 m × 0.32 mm × 0.27 m) silica capillary column (length: 30 m; internal diameter: 0.32 mm; Supelco, Bellefonte, USA) using helium as a carrier gas. Column initial temperature was set to 170 °C for 10 min, and then, it was raised to 220 °C at 2.5 °C per min and finally maintained at 215 °C for a further 5 min. FAMES were identified by comparison with previously characterized standards. Specific unclear peaks were identified by GLC-MS (TRACETM GC Ultra and PolarisQ mass spectrometer; Thermo Fisher Scientific, Spain) [38].

### 2.9. Statistical Analysis

Data are reported as mean ± standard deviation. Data were compared statistically using the analysis of variance, at a significance level of 5%. All variables were checked for normality and homogeneity of variance using the Kolmogorov–Smirnoff and the Levene’s tests, respectively [39]. Otherwise, an arcsin transformation was performed to attain normality. When arcsin-transformed data were not normally distributed, then Kruskall–Wallis non-parametric test was applied to the non-transformed data. An independent sample *t*-test was performed to compare sex steroid hormones, spawning quality, egg biochemical and fatty acid composition for commercial diet feeding phase to check the broodstock selection (HG or LG) effect. One way and two-way ANOVA were applied to the results of sex steroid hormones, sperm and egg and larval quality parameters (total eggs; fertilized eggs; viable eggs; hatched larvae; 3dph larvae per spawn per kg female and fertilization, egg viability, hatching and larval survival rates), egg biochemical and fatty acid composition of experimental diet feeding phase to determine the combined effects of broodstock selection (HG or LG) and diet (FO or VO) and the interaction of broodstock selection and diet. Pearson’s correlation coefficient and regression analysis were performed to check the relationship between spawning quality parameters. All data were analysed using the program IBM SPSS version 20 for Windows (IBM SPSS Inc., Armonk, NY, USA). 

## 3. Results

### 3.1. Phase I: Evaluation of Spawning Quality before Feeding the Experimental Diet

#### 3.1.1. Plasma Sex Steroid Hormones

Male and female plasma sex steroid hormone levels were not significantly different between HG and LG broodstock fed the same commercial diet (Table 4). The levels of plasma testosterone and 17β-estradiol were, respectively, 13 and 20% higher in HG than in LG female broodstock (Table 4). Plasma testosterone level was significantly higher in males than in females, being almost 2.9 and 3.3 folds higher in males than in females of HG or LG broodstock, respectively. Pearson’s correlation analysis result revealed that there was no correlation between broodstock body weight and steroid sex hormone levels (Supplementary Table S1). There was a strong positive correlation (r = 0.852; *p* = 0.001) between plasma testosterone and 11-ketotestosterone levels in males and, to a lower extent (r = 0.500; *p* = 0.05), between testosterone and 17β-estradiol levels in females. The GSI was significantly higher in HG males than in LG males (Table 4), whereas female GSI showed large deviations and no significant differences were found between HG and LG females for this parameter. No differences were found in HSI of males or females between HG and LG broodstock (Table 4), neither any relation between broodstock body weight and the foresaid parameters (Supplementary Table S2).

#### 3.1.2. Evaluation of Egg and Larval Quality

After one month of feeding the commercial diet (Phase I) at the beginning of the spawning season (December-January), there were no significant differences (*p* > 0.05) in reproductive performance in terms of fecundity (expressed per spawn and per kg female) between HG and LG broodstock (Table 5). The number of viable eggs/spawn /kg female was 15% higher for HG than for LG broodstock, showing the lowest *p* value, but still not significantly different (Table 5). Accordingly, in terms of larval output, HG broodstock produced relatively higher (15%) number of 3 dph larvae per spawn per kg female than the LG broodstock (Table 5). 

Spawning quality parameters, in terms of percentage of fertilization, hatching and larval survival, were not significantly different (*p* > 0.05) between HG and LG broodstock (Table 6). However, egg viability was significantly (*p* < 0.001) higher for HG than for LG broodstock (Table 6).

#### 3.1.3. Biochemical Analysis

The biochemical (Table 7) and fatty acid composition (Table 8) of the eggs also did not show significant (*p* > 0.05) differences between HG and LG broodstock fed with the commercial diet. The crude protein and crude lipid contents of eggs of HG or LG broodstock are 73% or 69% and 18% or 24% of dry matter, respectively (Table 7). The fatty acid profile of eggs from broodstock fed the commercial diet did not differ much between high and low growth broodstock (Table 8), and only 18:2n-9 (*p* = 0.005) and 18:3n-3 (*p* = 0.041) were respectively slightly lower and higher in HG than in LG eggs. Although they were all fed the same diet over the one-month period, all the individual LC-PUFA including EPA, DHA, ARA and, particularly, 20:3n-3 (*p* = 0.085) were slightly higher in the eggs from broodstock selected for high growth (HG) than in those from LG group (Table 8). Consequently, the total contents in PUFA from n-3 or n-6 series were respectively 18% and 5% higher in the eggs from broodstock selected for high growth (HG), whereas saturated and monounsaturated fatty acids tend to be lower than in those from LG group, nevertheless none of these tendencies were significant (*p* > 0.05) (Table 8). 

### 3.2. Phase II: Evaluation of Mass Spawning Quality after Feeding the Experimental Diets

#### 3.2.1. Plasma Sex Steroid Hormones

One-way ANOVA results indicated that plasma testosterone levels in LGFO and LGVO males were significantly (*p* = 0.033) higher than in HGVO broodstock (Table 9), whereas in females, LGFO and HGVO were lower in plasma testosterone (*p* = 0.004) than HGFO females (Table 9). Besides, 17β-estradiol levels in the LGFO females were significantly (*p* = 0.002) lower than in LGVO and HGVO (table 9). The two-way ANOVA analysis showed that male testosterone (*p* = 0.027) and 11-keto-testosterone (*p* = 0.029) levels were significantly higher in LG broodstock (Table 9), whereas female 17β-estradiol levels were significantly (*p* = 0.001) higher in broodstock fed the VO diet (Table 9). Moreover, there was a significant (*p* = 0.001) interaction between selection and diet in the female testosterone plasma levels that in HG broodstock were higher when fish was fed the FO diet, whereas the opposite trend could be found in LG broodstock (Table 9). Pearson’s correlation coefficient analysis showed that male 11-ketotestosterone levels were significantly negatively (r = −0.504; *p* = 0.05) correlated to broodstock body weight (Supplementary Table S3), following a linear relation (Supplementary Table S4), whereas female body weight did not show any relation to steroid hormone levels. There was also a significant positive correlation (r = 0.699; *p* = 0.002) between male testosterone and 11-ketotestosterone hormone levels. 

#### 3.2.2. Sperm Quality

Regarding sperm quality, sperm from the broodstock selected for high growth showed a significantly (*p* < 0.01) higher cell concentration (Table 10), regardless the diet fed, reflected in the strong effect of broodstock selection observed in the two-way ANOVA (Table 10). Whereas sperm viability was not affected by either broodstock selection or diet, sperm motility (95–98%) was significantly (*p* = 0.048) increased in the HG broodstock. On the contrary, sperm motility duration was increased by feeding both HG and LG broodstock with the VO diet (*p* < 0.001) (Table 10). Besides, the sperm motility was highly significantly correlated (r = 0.635; *p* = 0.015) to sperm concentration, and mildly correlated (r = 0.521; *p* = 0.056) to sperm viability (Supplementary Table S5). Besides, sperm concentration was slightly correlated (r = −0.544; *p* = 0.055) to plasma 11-ketotestosterone levels (ST. 5) followed a linear regression (R^2^ = 0.409; *p* = 0.018) (Supplementary Table S6). Moreover, sperm concentration followed a significant linear relationship with testosterone (R^2^ = 0.305; *p* = 0.050) (Supplementary Table S6), whereas other sperm parameters were not influenced by the male steroid hormones.

#### 3.2.3. Evaluation of Egg and Larval Quality

Fecundity in terms of total number of eggs produced per spawn per kg female was significantly (*p* < 0.001) highest for LGFO broodstock (Table 11). Therefore, the two-way ANOVA analysis denoted a significantly (*p* = 0.009) higher number of eggs produced per spawn per kg female in broodstock fed the FO diet and selected for LG, denoting the interaction between selection and diet (*p* = 0.005) (Table 11). The same results were also found for the number of fertilized eggs (Table 11). On the contrary, there was not a combined effect of selection and diet for the other fecundity parameters studied, which were all significantly (*p* < 0.001) improved in broodstock fed the FO diet, regardless the selection group (Table 11). Thus, the number of viable eggs hatchlings and larvae, per spawn per kg female was significantly higher for HGFO and LGFO than for LGVO or HGVO broodfish (Table 11). 

Regarding the relative spawning quality parameters, fertilization rates were significantly (*p* < 0.001) highest in HGFO eggs and lowest in LGVO eggs (Table 12), the two-way ANOVA showing the strong improvement in this parameter of either selection of HG broodstock (*p* = 0.019) or feeding FO diets (*p <* 0.001). Egg viability rate was even more strongly affected by the broodstock selection (*p* = 0.000) and broodstock diet (*p <* 0.001) (Table 12). Thus, the egg viability rate was high (93%) in HGFO broodstock, followed by LGFO (63.7%), whereas both LGVO (55%) and HGVO (54%) broodfish produced a significantly (*p <* 0.001) lower proportion of viable eggs (Table 12). The hatching and larval survival rates were found to be higher in FO diet fed broodstock irrespective of the selection criterion (Table 12). These findings clearly indicate that dietary fatty acids from FO source had strong positive influence on gilthead seabream broodstock reproductive performance, and high growth selected broodstock had higher proportions of fertilized and viable eggs. 

The estimated total number of eggs spawned by the total number of females per tank, or by a single female, or by kg body weight females was estimated, and the egg mass (g, wet weight) production per spawn per kg female was also calculated. The results indicated that gilthead seabream female broodstock (kg female) produces 2.18 to 2.88 million eggs and 0.58 to 1.12 million larvae (3dph) in 3 months spawning period (January to April). It was also noticed that HGFO broodstock (18 females) group produced relatively higher number of larvae (24.55 million) in the entire 3 months spawning season compared to other broodstock group. The larvae production per kg female was also relatively higher for HGFO broodstock (1.12 million). The egg mass production per spawn per kg female was found to be 9.5 to 12 g (mean egg wet weight ≈ 350 mg) for gilthead seabream broodstock. 

Pearson’s correlation coefficient analysis showed that sperm viability had a strong positive correlation (r = 0.997; *p* = 0.003) to egg fertilization rate and egg viability (r = 0.957; *p* = 0.043). There was also a positive correlation between egg fertilization rate and egg viability (r = 0.934; *p* = 0.066), as well as between egg hatching rate (r = 0.957; *p* = 0.043) and larval survival rate (Supplementary Table S7). We also found a significant linear relationship between sperm viability and fertilisation rate (R^2^ = 0.995; *p* = 0.003) and viability (R^2^ = 0.915; *p* = 0.043) percentage; sperm motility duration had a slight linear relation to egg fertilization rate (R^2^ = 0.863; *p* = 0.071) (Supplementary Table S8).

#### 3.2.4. Biochemical Analysis

Egg proximate composition was affected by either broodstock selection or dietary fatty acid profile (Table 13). The highest protein and moisture contents and the lowest lipid contents were found in the eggs from LGVO broodstock, that were significantly different from those of LGFO broodstock (Table 13). The two-way ANOVA analysis showed the significant effect of the VO diet, increasing egg protein and moisture contents and reducing lipid contents (Table 14), being significantly different in the eggs from LG broodstock but not in those from HG broodstock, due to the interaction between Diet and Selection (Table 13).

Egg fatty acid composition markedly reflected the broodstock diet (Table 14). Thus, feeding the VO diet significantly reduced the egg contents in 16:1n-7, 16:2n-4, 16:4n-3, 18:1n-7, 18:1n-5, 18:2n-4, 18:3n-4, 18:4n-3, 18:4n-1, 20:1n-9, 20:1n-7, 20:1n-5, 20:3n-9, 20:4n-6, 20:3n-3, 20:4n-3, 20:5n-3, 22:1n-11, 22:4n-6, 22:5n-6, 22:5n-3, n-3/n-6 and 20:3n-6/20:2n-6, whereas it increased the egg contents in 18:1n-9, 18:2n-6, 18:3n-3, 20:2n-6, n-6 FA, n-9 FA, DHA/ARA and DHA/EPA (Table 14). However, certain fatty acids were contrary to their dietary levels such as 18:2n-9 and 20:2n-6 that were increased in the eggs from VO fed broodstock, and the ratios 20:2n-9/20:1n-9 and 20:4n-3/20:3n-3 that were respectively increased and reduced. Finally, despite the marked dietary changes in 22:6n-3, the level of this fatty acid was not significantly different in eggs of the different broodstock. Finally, the effect of selection on the eggs fatty acid profiles was very mild, and limited the fish fed FO diet (Table 14). For instance, in the eggs from HGFO broodstock, the elongation product 20:2n-6 was increased in comparison to LGFO, whereas the desaturation ratios 16:4n-3, 18:4n-3, 18:4n-3/18:3n-3 were reduced (Table 14). 

## 4. Discussion

Genetic selection programmes with teleosts had focussed much on the improvement of growth rates, but in recent years, many other productive traits have been used [40]. The major traits used in selective breeding in fish include feed efficiency, skeletal deformities, disease resistance, fillet yield, and flesh and carcass quality [2,41,42]. In genomic selection, DNA maker-based information is used to predict the breeding value of different genotyped traits [43]. This approach has shown accurate prediction of breeding value for the growth trait as compared to conventional methods of selection and has been widely adopted in salmonids [44,45]. In recent times, the improvement of feed efficiency through genetic selection programs has also gained much attention [42,46]. Similarly, efforts towards selection of fish for better utilisation of plant-based diets have also been made [18,47,48]. It is also reported that feeding diets containing very low levels of fish meal and fish oil over the full life cycle, from early life to broodstock, in the gilthead seabream do not affect growth [49]. The latter authors unfortunately did not look into the reproductive performance of gilthead seabream fed such diets. A high nutritional plasticity has been reported in this protandrous hermaphroditic fish [18]. The above-mentioned studies with gilthead seabream focused on the growth traits and other physiological and metabolic parameters, without addressing whether broodstock selection for growth trait and the utilisation of fish oil or vegetable oil-based diets affect reproductive performance.

Marine teleosts show limited ability to bio-convert LC-PUFAs from LA and ALA substrate due to low expression of the *fads2* gene with a low activity of Fads enzyme [50,51]. This attribute can significantly affect the reproductive performance in fish, if sufficient amount of essential fatty acids is not supplied in the broodstock diet. There are few strategies applied to improve the spawning quality in fish under low fish meal and fish oil feeding regimes through broodstock nutritional programming [26,52,53]. We conducted the mass spawning of gilthead seabream broodstock of either high or low growth broodstock under two feeding regimes (FO or VO diet). The body weight of HG broodstock was significantly higher than that of the LG broodstock, well in conformity with the selection for growth trait, and we maintained an equal broodstock biomass for both HG and LG groups. Since the broodstock diet is known to have a strong influence on spawning quality in fish, the formulated broodstock diet in our experiment contained same level of ARA, EPA and DHA in the FO and VO diets used for gilthead seabream broodstock as in earlier studies [24,25,26,52]. The broodstock selected for high growth showed higher GSI, supporting the production of higher number of eggs by the HG broodstock under commercial diet feeding regime. Likewise, HSI was found to be higher for female broodstock in this study, which may suggest the higher requirement of vitellogenin synthesis in the female liver for the production of lipoproteins, particularly phosphovitine and lipovitellin rich in n-3 LC-PUFA, which transport lipids to the developing oocyte. These results agree with the higher GSI observed in female in comparison to males as reported earlier [54,55]. Testosterone, a major hormone which regulates the spermatogenesis process from spermatogonial proliferation to spermatocyte formation in fish through endocrine pathway [56], was significantly higher in male HG broodstock. Plasma steroid hormone levels of both male and female gilthead seabream showed values as reported in other studies [57,58].

11-ketotestosterone is the main androgen, controlling spermatogenesis and also secondary sexual characteristics in males [59,60]. 11KT is synthesised from testosterone, and our data show a strong positive correlation between plasma testosterone and 11KT levels in male seabream broodstock. Moreover, in the females, we found plasma testosterone and 17β-estradiol levels had a slight correlation, as 17β-estradiol production is dependent on testosterone as a substrate [61,62,63]. The broodstock size had no relation to the plasma steroid levels in gilthead seabream broodstock as reported earlier in rainbow trout [64] or Atlantic salmon [65]. The HG broodstock always had a relatively high spawning quality. This is in agreement with data from other studies where it is observed that bigger size broodstock produces higher number of eggs and larvae in fish as reported in seabream [66], channel catfish [67], rainbow trout [68], Atlantic salmon [69] and African catfish [70]. 

The fatty acid composition of broodstock diet is known to play a significant role in determining egg and larval quality in fish [52,71]. We observed few spawning quality parameters to be of similar magnitude in both HG or LG broodstock. This might be due to the feeding with similar fatty acid composition diet in the commercial diet study period. Female steroid hormone 17β-estradiol level was significantly improved by the experimental diet as compared to commercial diet. A high dietary supply of fatty acid precursors increases the conversion of ALA to n-3 LC-PUFA promoted by oestrogen in pregnant women, claimed to be important to fulfil the essential fatty acid requirements of foetus and neonate [72].

Although less well studied, it is known since long that that sperm quality is also a very important variable in broodstock management in fish, with a strong influence on egg fertilisation rate [56,73]. In general, the sperm quality increases with age and size of fish and reported maximum quality to certain sizes, and further increase in age or size leads to reduction in sperm quality [74,75]. In rainbow trout, it is shown that two to three year old broodstock has good sperm quality and beyond this age, the sperm quality is drastically decreased [74]. We also found that sperm cell concentration of seabream broodstock was significantly higher in the higher growth/bigger sized than in the lower growth/smaller sized broodstock. In some terrestrial animals, sperm cell concentration is reported to have a positive correlation to sperm motility [76] as observed in our study. The sperm viability is directly influenced by sperm motility duration, which helps the sperm to search for the eggs to enter through egg micropyle and fuses with the oocyte plasma membrane to fertilize the eggs [56,77,78]. Our data showed that in seabream broodstock, sperm viability significantly increased egg fertilisation and viability rate generally observed in fish [79,80], although such a relation was not observed in some species such as the Atlantic salmon [81], sockeye salmon [82] or Atlantic cod [83]. The dietary fatty acid composition influences the sperm quality in fish [84,85,86,87]. In this study, we observed that sperm quality is not much affected by the parental diet fatty acid profile. This may be that the level of essential fatty acids (EFAs) present in both FO and VO diets was sufficient enough for normal spermatogenesis in gilthead seabream [26]. The dietary ARA, EPA and DHA levels used here were found to be more or less at the same level than in studies with other fish species such as European sea bass [88], Senegalese sole [86], rainbow trout [89], European eel [90], Siberian sturgeon [91] or Eurasian perch [92]. 

The continuous spawners like gilthead seabream have very short vitellogenic periods, and the spawning quality is directly affected by the parental dietary fatty acid composition [19,52,55]. It has been suggested that the biochemical composition of fish eggs is related to the spawning quality as egg reserves must satisfy embryonic development [93]. Gilthead seabream females continue to actively feed during sexual maturation throughout the spawning season and produce an egg biomass greater than their own body weight, which require greater amount of EFAs during the spawning period [19,52]. DHA, as an EFA, plays a more important role in the enzyme activity of the cell membrane and in physiological balance than EPA. Deficiencies in DHA could lead to reduction in egg and larval quality [71]. Study confirmed for gilthead seabream that larvae had preferentially conserved DHA over EPA during deprivation, which indicates essentiality of DHA in the parental diet [94]. In our study also, we observed that FO diet had higher DHA and EPA than VO diet. In turn, the higher DHA and EPA of parental FO diet significantly improved the spawning quality in seabream broodstock as reported in the above studies. 

Moreover, DHA content in the eggs was effectively increased in females fed with FO diet and also broodstock selected for high growth had relatively higher amount of DHA than LG broodstock. It indicates an efficient bioconversion or specific retention of fatty acids from diet to eggs as seen in many fish. The reduced DHA, EPA and ARA levels in the VO diet led to a significant reduction in number of egg and larval production in gilthead seabream. In many species, egg viability is an ideal indicator to ascertain the egg quality. It was observed that broodstock selected for high growth and fed with FO diet (HGFO, 81%) had produced 27% more viable eggs as compared to low growth broodstock fed with VO diet (LGVO, 54%). This is a strong evidence that indicate that a low level of EFAs in parental diet reduces the egg and larval viability [52]. The higher DHA/EPA ratios in the eggs also suggest the activation of the Sprecher pathway to synthesise DHA after beta-oxidation from 24:6n-3 [95]. Increase in egg DHA content by FO diet in females would lead to an increased production of docosanoids, which also play an important role in induction of oocyte maturation, improving fecundity in terms of eggs produced [96]. FO replacement by combination of linseed and rapeseed oil in parental diet reduced the amount of EFAs and increase in 18:2n-6 and 18:3n-3 precursors, which tailor the gilthead seabream offspring to produce juveniles to better utilize the low FM and FO diets [24]. Additionally, it was noticed that gilthead seabream broodstock selected for high growth certainly has an improved spawning quality particularly when the broodstock is fed with sufficient levels of essential fatty acids.

## 5. Conclusions

This study emphasizes the strong positive effect of dietary fatty acids on the reproductive performance, egg and larval quality of gilthead seabream broodstock. The high growth (HG) trait gilthead seabream broodstock was found to produce higher number of sperm cells and had increased sperm motility. This group had significantly higher egg viability percentage, which ultimately produced relatively higher number of eggs and larvae. The steroid hormone production, sperm and egg quality was markedly improved in the broodstock selected for high growth and fed with fish oil-based diet. The egg viability and number of eggs and larvae production were also significantly improved by the dietary fatty acid of FO diet and to some extent by broodstock selection. This study clearly indicates that gilthead seabream broodstock selected on growth trait could have positive role in improvement of sperm and egg quality to produce viable progeny.

## Figures and Tables

**Table 1:** Description of gilthead sea bream broodstock selected for mass spawning experiment.

Broodstock Details	HGFO	HGVO	LGFO	LGVO	One-Way ANOVA *p* Value
Broodstock density/tank					
Male (*n*)	32	33	27	27	
Female (*n*)	18	18	22	22	
Total (*n*)	50	51	49	49	
Broodstock biometry					
Male length (cm)	35.0 ± 2.7	35.3 ± 2.2	33.8 ± 3.1	34.2 ± 3.0	0.135
Male weight (kg)	1.0 ± 0.3 ^ab^	1.1 ± 0.2 ^a^	0.9 ± 0.2 ^b^	0.9 ± 0.3 ^ab^	0.015
Female length (cm)	35.8 ± 4.3 ^a^	35.2 ± 3.2 ^a^	32.3 ± 1.5 ^b^	32.8 ± 1.8 ^b^	<0.001
Female weight (kg)	1.2 ± 0.5 ^a^	1.2 ± 0.6 ^a^	0.8 ± 0.1 ^b^	0.8 ± 0.1 ^b^	<0.001
Male biomass (kg)	33	35	24	25	
Female biomass (kg)	22	21	17	18	
Total biomass (kg)	55	57	41	43	
Female/male biomass	1.5	1.7	1.4	1.4	

Different superscripts in each row would indicate significant differences among broodfish groups for a given parameter (*p* < 0.05, One-way ANOVA, Tukey Post-Hoc).

**Table 2:** Ingredients and proximate composition of the broodstock experimental diets used for mass spawning study.

Feed Ingredients (%)	FO Diet	VO Diet
Fish meal, North-Atlantic	57.33	57.33
Krill meal	7.00	7.00
Squid meal	3.00	3.00
Wheat	21.99	21.99
Fish oil, South American	9.96	0.00
Rapeseed oil	0.00	8.45
Linseed oil	0.00	1.50
Vitamin-Mineral premix	0.50	0.50
Astaxanthin 10%	0.03	0.03
L-Histidine HCl	0.20	0.20
**Proximate composition**		
Crude protein (% dry matter, DM)	51.7	51.2
Crude lipid (% DM)	16.8	16.5
Ash (% DM)	10.6	10.4
Moisture (%)	8.4	8.9

**Table 3:** Fatty acid profiles (% total fatty acids) of the fish oil (FO) and vegetable oil (VO) broodstock diets.

%TFA	FO Diet	VO Diet
14:0	3.44	2.17
14:1n-7	0.03	0.03
14:1n-5	0.11	0.08
15:0	0.30	0.16
15:1n-5	0.02	0.03
16:0 ISO	0.06	0.03
16:0	13.29	9.18
16:1n-7	5.12	2.38
16:1n-5	0.22	0.09
16:2n-6	0.01	0.00
16:2n-4	0.61	0.19
17:0	0.67	0.10
16:3n-4	0.13	0.15
16:3n-3	0.13	0.07
16:3n-1	0.08	0.05
16:4n-3	0.93	0.28
16:4n-1	0.03	0.02
18:0	3.54	2.53
18:1n-9	12.72	35.07
18:1n-7	2.93	2.79
18.1n-5	0.18	0.12
18:2n-9	0.04	0.02
18.2n-6	4.56	13.49
18:2n-4	0.23	0.05
18:3n-6	0.27	0.00
18:3n-4	0.15	0.07
18:3n-3	1.59	9.58
18.3n-1	0.03	0.01
18:4n-3	2.14	1.01
18:4n-1	0.14	0.03
20:0	0.52	0.49
20:1n-9	0.47	0.24
20:1n-7	3.88	3.19
20.1n-5	0.34	0.12
20:2n-9	0.05	0.02
20:2n-6	0.23	0.14
20:3n-9	0.09	0.04
20:3n-6	0.12	0.04
20:4n-6	1.00	0.28
20:3n-3	0.12	0.06
20:4n-3	0.67	0.21
20:5n-3	14.97	4.81
22:1n-11	5.20	3.20
22:1n-9	0.73	0.64
22:4n-6	0.14	0.06
22:5n-6	0.33	0.09
22:5n-3	1.91	0.36
22:6n-3	15.56	6.23
Total saturates	21.75	14.63
Total monoenes	31.93	47.98
Total n-3	38.01	22.62
Total n-6	6.659	14.10
Total n-9	14.09	36.03
Sum n-3 LC-PUFA	33.23	11.67
EPA/ARA	15.02	17.41
ARA/EPA	0.067	0.057
DHA/EPA	1.039	1.296
DHA/ARA	15.62	22.57
n-3/n-6	5.71	1.60

**Table 4:** Hepatosomatic, gonadosomatic index, steroid sex hormone levels of gilthead seabream male or female broodstock of high (HG) or low growth (LG) groups before feeding the experimental diets. Male-HG (*n* = 6); Male-LG (*n* = 7); Female-HG (*n* = 6) and Female-LG (*n* = 5).

Broodstock Sex	Parameters	High Growth (HG)	Low Growth (LG)	*t*-Test (*p* Value)
**Male**	Testosterone (ng/mL)	2.3 ± 0.8	2.3 ± 1.4	0.989
	11 Keto-testosterone (ng/mL)	0.2 ± 0.1	0.4 ± 0.3	0.243
	GSI%	7.8 ± 2.5 ^a^	2.0 ± 2.4 ^b^	0.001
	HSI%	1.0 ± 0.2	1.5 ± 0.9	0.211
**Female**	Testosterone (ng/mL)	0.8 ± 0.2	0.7 ± 0.4	0.649
	17β-estradiol (ng/mL)	1.0 ± 0.3	0.8 ± 0.3	0.337
	GSI%	6.3 ± 9.7	5.1 ± 4.8	0.805
	HSI%	1.7 ± 0.4	1.4 ± 0.4	0.236

Different superscripts in each row indicate significant differences among HG or LG broodfish groups (*p* < 0.05, independent sample *t*-test). GSI (%) (gonadosomatic index) = (Gonad weight, g/weight of fish, g) × 100; HSI (%) (hepatosomatic index) = (Liver weight, g/weight of fish, g) × 100.

**Table 5:** Reproductive performance (fecundity) of gilthead seabream broodstock selected for high (HG) or low (LG) growth before experimental diet feeding period (HG, *n* = 56; LG, *n* = 54).

Fecundity Parameters	High Growth (HG)	Low Growth (LG)	*t*-Test (*p* Value)
Total number of eggs/spawn/kg female	21,534 ± 15,024	20,549 ± 15,430	0.735
Fertilized eggs/spawn/kg female	21,114 ± 14,749	20,114 ± 15,242	0.727
Viable eggs/spawn/kg female	16,141 ± 11,079	13,689 ± 12,319	0.275
Hatched larvae/spawn/kg female	15,872 ± 10,389	13,744 ± 10,773	0.340
Larval survival 3dph/spawn/kg female	12,644 ± 8804	10,762 ± 9560	0.334

**Table 6:** Relative spawning quality (%) in gilthead seabream broodstock selected for high (HG) or low (LG) growth before experimental diet feeding period (HG, *n* = 56; LG, *n* = 54).

Spawning Quality Parameters	High Growth (HG)	Low Growth (LG)	*t*-Test (*p* Value)
Fertilization %	96.4 ± 8.9	96.7 ± 4.5	0.617
Egg viability %	75.4 ± 16.6 ^a^	64.6 ± 24 ^b^	0.014
Hatching %	92.2 ± 7.7	91 ± 12.1	0.762
Larval survival (3dph) %	80.2 ± 14.3	78.4 ± 17.1	0.731

Means bearing different superscript letters differ significantly (*p* < 0.05, Independent sample *t*-test).

**Table 7:** Biochemical composition of eggs obtained from the gilthead seabream broodstock selected for high (HG) or low (LG) growth before feeding broodstock experimental diets (HG, *n* = 3; LG, *n* = 3).

Biochemical Composition	High Growth (HG)	Low Growth (LG)	*t*-Test (*p* Value)
Crude protein (% DM)	72.6 ± 5.3	68.7 ± 3.6	0.171
Crude lipid (% DM)	18.4 ± 7.9	23.5 ± 0.8	0.146
Ash (% DM)	2.7 ± 1.2	3.6 ± 0.3	0.148
Moisture (%)	88 ± 0.5	87.7 ± 0.3	0.213

**Table 8:** Fatty acid composition (% total fatty acids) of eggs of gilthead sea bream broodstock selected for high (HG) or low (LG) growth before feeding the experimental diets (HG, n = 2; LG, n = 2).

Fatty Acid (%TFA)	High Growth (HG)	Low Growth (LG)	*t*-Test (*p* Value)
14:0	1.54 ± 0.29	1.55 ± 0.49	0.980
14:1n-7	0.01 ± 0.01	0.01	0.391
14:1n-5	0.07 ± 0.02	0.07 ± 0.02	0.834
15:0	0.23 ± 0.03	0.24 ± 0.07	0.696
16:0 ISO	0.08 ± 0.08	0.09 ± 0.08	0.896
16:0	14.00 ± 0.89	16.23 ± 3.34	0.246
16:1n-7	3.10 ± 0.58	3.71 ± 0.53	0.175
16:1n-5	0.08 ± 0.03	0.08 ± 0.01	1.000
16:2n-4	0.15 ± 0.02	0.14 ± 0.01	0.267
17:0	0.14 ± 0.01	0.14 ± 0.02	0.628
16:3n-4	0.25 ± 0.02	0.26 ± 0.04	0.414
16:3n-3	0.08 ± 0.01	0.09 ± 0.02	0.304
16:3n-1	0.08 ± 0.02	0.09 ± 0.02	0.344
16:4n-3	0.07 ± 0.02	0.07 ± 0.02	0.613
18:0	3.80 ± 0.50	4.45 ± 1.05	0.320
18:1n-9	27.78 ± 2.24	29.59 ± 4.46	0.506
18:1n-7	2.71 ± 0.39	2.80 ± 0.45	0.770
18:1n-5	0.13 ± 0.01	0.15 ± 0.02	0.211
18:2n-9	0.19 ± 0.02 ^b^	0.24 ± 0.01 ^a^	0.005
18.2n-6	12.29 ± 0.51	12.01 ± 1.07	0.655
18:2n-4	0.13 ± 0.01	0.13 ± 0.01	1.000
18:3n-6	0.28 ± 0.03	0.31 ± 0.01	0.161
18:3n-4	0.16 ± 0.03	0.15 ± 0.04	0.704
18:3n-3	2.72 ± 0.16 ^a^	2.41 ± 0.18 ^b^	0.041
18:4n-3	0.47 ± 0.05	0.38 ± 0.11	0.217
18:4n-1	0.09 ± 0.01	0.07 ± 0.02	0.124
20:0	0.09 ± 0.01	0.10 ± 0.03	0.758
20:1n-9	0.16 ± 0.03	0.16 ± 0.03	0.824
20:1n-7	0.98 ± 0.14	0.98 ± 0.21	0.970
20.1n-5	0.12 ± 0.02	0.12 ± 0.03	0.879
20:2n-9	0.09 ± 0.00	0.09 ± 0.01	0.705
20:2n-6	0.47 ± 0.04	0.45 ± 0.07	0.681
20:3n-6	0.22 ± 0.01	0.21 ± 0.02	0.439
20:4n-6	0.68 ± 0.03	0.64 ± 0.08	0.418
20:3n-3	0.34 ± 0.02	0.31 ± 0.03	0.085
20:4n-3	0.73 ± 0.06	0.61 ± 0.15	0.182
20:5n-3	4.39 ± 0.54	3.63 ± 1.42	0.378
22:1n-11	0.19 ± 0.02	0.20 ± 0.05	0.620
22:1n-9	0.09 ± 0.01	0.10 ± 0.03	0.656
22:4n-6	0.08 ± 0.01	0.07 ± 0.01	0.320
22:5n-6	0.22 ± 0.02	0.19 ± 0.06	0.396
22:5n-3	2.30 ± 0.34	1.78 ± 0.78	0.289
22:6n-3	18.22 ± 3.43	14.90 ± 7.97	0.486
Total Saturates	19.80 ± 1.20	22.69 ± 4.56	0.265
Total Monoenes	35.43 ± 2.74	37.96 ± 5.62	0.459
Total n-3	29.31 ± 4.29	24.19 ± 10.52	0.418
Total n-6	14.22 ± 0.51	13.87 ± 1.09	0.574
Total n-9	28.34 ± 2.26	30.20 ± 4.53	0.500
Total n-3 LC-PUFA	25.98 ± 4.30	21.24 ± 10.31	0.443
EPA + DHA	22.61 ± 3.92	18.53 ± 9.38	0.467
ARA/EPA	0.16 ± 0.03	0.20 ± 0.06	0.307
EPA/ARA	6.50 ± 0.99	5.54 ± 1.65	0.359
DHA/ARA	27.01 ± 5.62	22.50 ± 10.19	0.476
DHA/EPA	4.14 ± 0.42	3.91 ± 0.68	0.596
DHA/DPA	7.89 ± 0.39	8.06 ± 1.02	0.775
n-3/n-6	2.07 ± 0.37	1.79 ± 0.86	0.577
n-6/n-3	0.49 ± 0.09	0.69 ± 0.34	0.346
20:2n-9/20:1n-9	0.57 ± 0.08	0.60 ± 0.07	0.579
18:3n-6/18:2n-6	0.02 ± 0.00	0.03 ± 0.01	0.182
20:3n-6/20:2n-6	0.47 ± 0.05	0.47 ± 0.08	0.959
18:4n-3/18:3n3	0.17 ± 0.02	0.16 ± 0.03	0.543
20:4n-3/20:3n-3	2.15 ± 0.23	1.96 ± 0.42	0.453

Means bearing different superscript letters differ significantly (*p* < 0.05, Independent sample *t*-test). The sample size of *n* = 2 is taken for the fatty acid analysis of egg samples of different broodstock groups.

**Table 9:** Steroid sex hormone levels of gilthead seabream male or female broodstock of high (HG) or low growth (LG) groups fed with either FO or VO diet over three months of reproductive season (HGFO, *n* = 5; HGVO, *n* = 5; LGFO, *n* = 5; LGVO, *n* = 5).

Broodstock Sex	Steroid Sex Hormone	Broodfish Groups				One-Way ANOVA (*p* Values)	Two-Way ANOVA (*p* Values)		
		HGFO	HGVO	LGFO	LGVO		Selection	Diet	S × D
Male	Testosterone (ng/mL)	2.2 ± 0.2 ^ab^	1.2 ± 0.4 ^b^	2.9 ± 0.4 ^a^	2.8 ± 0.4 ^a^	0.033	0.027	0.383	0.561
	11 Keto-testosterone (ng/mL)	0.2 ± 0.01	0.04 ± 0.01	0.1 ± 0.04	0.1 ± 0.01	0.085	0.029	0.256	0.616
Female	Testosterone (ng/mL)	2.5 ± 0.4 ^a^	1.1 ± 0.2 ^b^	1.1 ± 0.1 ^b^	1.6 ± 0.2 ^ab^	0.004	0.074	0.089	0.001
	17β-estradiol (ng/mL)	1.8 ± 0.4 ^ab^	2.8 ± 0.4 ^a^	0.8 ± 0.1 ^b^	2.6 ± 0.3 ^a^	0.002	0.075	0.001	0.285

Different superscripts in each row would indicate significant differences among broodfish groups for a given parameter (*p* < 0.05, One-way ANOVA, Tukey Post-Hoc).

**Table 10:** Sperm quality of gilthead seabream broodstock selected for high (HG) or low (LG) growth fed with either FO or VO diet over three months of reproductive season (HGFO, *n* = 4; HGVO, *n* = 4; LGFO, *n* = 5; LGVO, *n* = 5).

Sperm Quality Parameters	Broodfish Groups				One-Way ANOVA (*p* Values)	Two-Way ANOVA (*p* Values)		
	HGFO	HGVO	LGFO	LGVO		Selection	Diet	S × D
Sperm concentration (10^9^ cells/mL)	9.8 ± 0.7 ^a^	10.3 ± 0.9^a^	3.9 ± 0.8 ^b^	5.4 ± 1.6 ^b^	0.0001	<0.001	0.210	0.512
Sperm viability %	86.4 ± 5.3	91.3 ± 7.7	89.2 ± 6.6	92.8 ± 1.8	0.5012	0.507	0.202	0.847
Sperm motility %	95.0 ± 3.5	98.8 ± 1.8	75 ± 15.5	91.0 ± 5.2	0.0504	0.048	0.140	0.344
Sperm motility duration (Seconds)	751 ± 26 ^b^	1287 ± 314 ^a^	777 ± 172 ^b^	1764 ± 184 ^a^	<0.0001	0.047	<0.0001	0.070

Means bearing different superscript letters differ significantly (*p* < 0.05, One-way ANOVA, Tukey Post-Hoc).

**Table 11:** Reproductive performance (fecundity) of gilthead seabream broodstock selected for high (HG) or low (LG) growth fed with either FO or VO diet over three months of reproductive season (HGFO, *n* = 70; HGVO, *n* = 67; LGFO, *n* = 72; LGVO, *n* = 72).

Fecundity Parameters	Broodfish Groups				One-Way ANOVA (*p* Values)	Two-Way ANOVA (*p* Values)		
	HGFO	HGVO	LGFO	LGVO		Selection	Diet	S × D
Total number of eggs/spawn/kg female	28,142 ± 10,552 ^b^	28,451 ± 10,502 ^b^	34,478 ± 11,100 ^a^	27,145 ± 12,667 ^b^	<0.001	0.062	0.009	0.005
Fertilized eggs/spawn/kg female	27,946 ± 10,493 ^b^	28,100 ± 10,479 ^b^	34,140 ± 11,083 ^a^	26,746 ± 12,643 ^b^	<0.001	0.072	0.007	0.005
Viable eggs/spawn/kg female	22,853 ± 9854 ^a^	15,326 ± 6631 ^b^	21,868 ± 9233 ^a^	14,767 ± 8589 ^b^	<0.001	0.457	<0.001	0.837
Hatched larvae/spawn/kg female	21,546 ± 9493 ^a^	13,407 ± 6317 ^b^	20,351 ± 8895 ^a^	13,650 ± 8276 ^b^	<0.001	0.633	<0.001	0.472
Larval survival 3dph/spawn/kg female	16,333 ± 9274 ^a^	8707 ± 5745 ^b^	14,793 ± 7956 ^a^	9100 ± 7012 ^b^	<0.001	0.529	<0.001	0.289

Means bearing different superscript letters differ significantly (*p* < 0.05 by One-way ANOVA, Tukey Post-Hoc).

**Table 12:** Relative spawning quality (%) in gilthead seabream broodstock selected for high (HG) or low (LG) growth fed with either FO or VO diet over three months of reproductive season (HGFO, *n* = 70; HGVO, *n* = 67; LGFO, *n* = 72; LGVO, *n* = 72).

Spawning Quality Parameters	Broodfish Groups				One-Way ANOVA (*p* Values)	Two-Way ANOVA (*p* Values)		
	HGFO	HGVO	LGFO	LGVO		Selection	Diet	S × D
Fertilization %	99.4 ± 1.7 ^a^	98.7 ± 2.3 ^b^	99 ± 1.8 ^ab^	98.4 ± 2.8 ^b^	<0.001	0.019	<0.001	0.397
Egg viability %	81.3 ± 15.6 ^a^	54.3 ± 13.6 ^c^	63.7 ± 18.1 ^b^	54.9 ± 15.7 ^c^	<0.001	0.000	<0.001	0.000
Hatching %	93.6 ± 9.7 ^a^	87.2 ± 13.4 ^b^	92.6 ± 9.4 ^a^	90.6 ± 10.7 ^ab^	<0.001	0.392	<0.001	0.037
Larval survival (3dph) %	75.3 ± 20.6 ^a^	64.4 ± 23.1 ^b^	73.1 ± 20.6 ^ab^	67.1 ± 22 ^b^	0.003	0.863	<0.001	0.210

Means bearing different superscript letters differ significantly (*p* < 0.05 by One-way ANOVA, Tukey Post-Hoc).

**Table 13:** Biochemical composition of eggs obtained from the gilthead seabream broodstock selected for high (HG) or low (LG) growth fed with either FO or VO diet over three months of reproductive season (HGFO, *n* = 3; HGVO, *n* = 3; LGFO, *n* = 3; LGVO, *n* = 3).

Egg Biochemical Composition	Broodfish Groups				One-Way ANOVA (*p* Values)	Two-Way ANOVA (*p* Values)		
	HGFO	HGVO	LGFO	LGVO		Selection	Diet	S × D
Crude protein (%DM)	70.2 ± 0.4 ^ab^	69.6 ± 2.6 ^b^	69.3 ± 1.6 ^b^	75.7 ± 1.1 ^a^	0.016	0.062	0.044	0.023
Crude lipid (%DM)	23.8 ± 1.2 ^a^	22.9 ± 0.7 ^a^	23.8 ± 0.9 ^a^	16.4 ± 0.8 ^b^	<0.0001	0.003	0.001	0.002
Ash (%DM)	3.5 ± 0.3	3.6 ± 0.2	3.3 ± 0.2	3.6 ± 0.5	0.763	0.902	0.605	0.934
Moisture (%)	87.7 ± 0.3 ^b^	87.7 ± 0.3 ^b^	87.4 ± 0.1 ^b^	89.2 ± 0.1 ^a^	<0.0001	0.003	<0.001	0.001

Means bearing different superscript letters differ significantly (*p* < 0.05, One-way ANOVA, Tukey Post-Hoc).

**Table 14:** Fatty acid composition (% total fatty acids) of eggs of gilthead sea bream broodstock selected for high (HG) or low (LG) growth and fed with either FO or VO diet over three months of reproductive season (n = 2 for all the broodstock group).

Fatty Acid (%TFA)	HGFO	HGVO	LGFO	LGVO	One-Way ANOVA (*p* Value)	Two-Way ANOVA (*p* Value)		
						Selection	Diet	S × D
14:0	2.44 ± 0.32	1.48 ± 0.19	2.66 ± 1.04	1.64 ± 0.46	0.271	0.676	0.079	0.947
14:1n-7	0.02 ± 0.00	0.01 ± 0.00	0.02 ± 0.01	0.03 ± 0.03	0.606	0.507	0.820	0.292
14:1n-5	0.1 ± 0.02	0.05 ± 0.00	0.1 ± 0.03	0.07 ± 0.03	0.242	0.479	0.079	0.664
15:0	0.28 ± 0.06	0.18 ± 0.01	0.27 ± 0.07	0.19 ± 0.04	0.210	0.946	0.056	0.840
15:1n-5	0.01 ± 0.00	0.02 ± 0.01	0.02 ± 0.01	0.02 ± 0.01	0.734	0.460	0.460	1.000
16:0 ISO	0.06 ± 0.01	0.03 ± 0.00	0.05 ± 0.01	0.04 ± 0.01	0.136	0.756	0.040	0.374
16:0	16.79 ± 3.61	13.01 ± 0.71	15.28 ± 1.42	13.53 ± 0.04	0.337	0.741	0.119	0.507
16:1n-7	5.45 ± 0.9 ^ab^	3.09 ± 0.31 ^bc^	6.1 ± 0.53 ^a^	2.97 ± 0.47 ^c^	0.013	0.561	0.003	0.410
16:1n-5	0.11 ± 0.01	0.07 ± 0.00	0.1 ± 0.04	0.07 ± 0.01	0.306	0.733	0.094	0.733
16:2n-4	0.37 ± 0.01 ^a^	0.13 ± 0.01 ^b^	0.47 ± 0.06 ^a^	0.13 ± 0.01 ^b^	0.001	0.103	<0.001	0.131
17:0	0.28 ± 0.01 ^b^	0.12 ± 0.00 ^c^	0.36 ± 0.02 ^a^	0.1 ± 0.00^c^	<0.001	0.019	<0.001	0.003
16:3n-4	0.28 ± 0.06	0.21 ± 0.01	0.27 ± 0.04	0.2 ± 0.01	0.202	0.720	0.054	1.000
16:3n-3	0.13 ± 0.02	0.07 ± 0.01	0.11 ± 0.01	0.07 ± ±0.00	0.041	0.507	0.010	0.507
16:3n-1	0.1 ± 0.03	0.07 ± 0.01	0.08 ± 0.01	0.06 ± 0.02	0.229	0.249	0.102	0.595
16:4n-3	0.15 ± 0.01 ^b^	0.07 ± 0.00 ^c^	0.25 ± 0.01 ^a^	0.07 ± 0.00 ^c^	0.000	0.000	<0.001	0.000
18:0	5.44 ± 1.2	3.93 ± 0.09	4 ± 0.45	4 ± 0.04	0.189	0.206	0.170	0.172
18:1n-9	20.29 ± 3.68 ^ab^	28.09 ± 1.62 ^a^	18.26 ± 0.95 ^b^	28.73 ± 1.73 ^ab^	0.020	0.685	0.004	0.446
18:1n-7	3.58 ± 0.64	2.57 ± 0.01	3.81 ± 0.35	2.71 ± 0.23	0.073	0.534	0.018	0.863
18:1n-5	0.21 ± 0.04	0.13 ± 0.00	0.18 ± 0.03	0.14 ± 0.01	0.077	0.570	0.021	0.407
18:2n-9	0.14 ± 0.01	0.2 ± 0.03	0.14 ± 0.02	0.19 ± 0.01	0.065	0.506	0.016	0.733
18.2n-6	6.69 ± 0.74 ^b^	11.81 ± 0.65 ^a^	5.79 ± 0.2 ^b^	12.11 ± 0.74 ^a^	0.001	0.537	<0.001	0.247
18:2n-4	0.27 ± 0.04 ^a^	0.1 ± 0.01 ^b^	0.28 ± 0.02 ^a^	0.08 ± ±0.00 ^b^	0.001	0.874	<0.001	0.446
18:3n-6	0.22 ± 0.04	0.25 ± 0.04	0.23 ± 0.01	0.24 ± 0.00	0.636	1.000	0.273	0.638
18:3n-4	0.26 ± 0.03 ^a^	0.13 ± 0.02 ^b^	0.26 ± 0.04 ^a^	0.12 ± 0.00 ^b^	0.008	0.791	0.002	1.000
18:3n-3	1.51 ± 0.01 ^a^	4.96 ± 0.76 ^b^	1.44 ± 0.02 ^a^	5.58 ± 0.79 ^b^	0.003	0.522	0.001	0.422
18:4n-3	0.87 ± 0.16 ^ab^	0.53 ± 0.07 ^b^	1.21 ± 0.06 ^a^	0.56 ± 0.06 ^b^	0.006	0.054	0.002	0.080
18:4n-1	0.2 ± 0.04 ^a^	0.08 ± 0.01 ^b^	0.25 ± ±0.00 ^a^	0.07 ± 0.01 ^b^	0.003	0.233	0.001	0.161
20:0	0.14 ± 0.04	0.07 ± 0.00	0.06 ± 0.08	0.08 ± 0.00	0.432	0.356	0.497	0.251
20:1n-9	0.24 ± 0.04 ^a^	0.13 ± 0.01 ^b^	0.22 ± 0.01 ^a^	0.13 ± 0.01 ^b^	0.012	0.468	0.003	0.468
20:1n-7	1.11 ± 0.18	0.86 ± 0.04	0.97 ± 0.06	0.87 ± 0.02	0.158	0.405	0.058	0.341
20:1n-5	0.2 ± 0.03 ^a^	0.1 ± 0.00 ^b^	0.18 ± 0.00^a^	0.1 ± 0.00 ^b^	0.004	0.374	0.001	0.374
20:2n-6	0.27 ± 0.04 ^ab^	0.34 ± 0.02 ^a^	0.21 ± 0.01 ^b^	0.32 ± 0.01 ^a^	0.013	0.074	0.004	0.223
20:3n-9	0.05 ± 0.01 ^ab^	0.03 ± 0.01 ^b^	0.06 ± 0.00 ^a^	0.03 ± 0.00 ^b^	0.007	0.047	0.002	0.230
20:3n-6	0.18 ± 0.00	0.17 ± 0.01	0.17 ± 0.01	0.17 ± 0.00	0.702	0.519	0.519	0.519
20:4n-6	1.04 ± 0.11 ^a^	0.6 ± 0.04 ^b^	0.94 ± 0.01 ^a^	0.54 ± 0.02 ^b^	0.002	0.116	0.001	0.693
20:3n-3	0.20 ± 0.01 ^b^	0.33 ± 0.01 ^a^	0.17 ± 0.01 ^b^	0.34 ± 0.01 ^a^	<0.001	0.452	<0.001	0.124
20:4n-3	1.00 ± 0.15 ^a^	0.65 ± 0.04 ^b^	1.13 ± 0.06 ^a^	0.65 ± 0.01 ^b^	0.008	0.306	0.002	0.306
20:5n-3	8.52 ± 2.55	5.14 ± 0.46	10.3 ± 1.24	5.07 ± 0.25	0.051	0.449	0.013	0.415
22:1n-11	0.35 ± 0.04 ^a^	0.17 ± 0 ^b^	0.32 ± 0.04 ^a^	0.17 ± 0.01 ^b^	0.006	0.434	0.001	0.434
22:1n-9	0.13 ± 0.01	0.11 ± 0.03	0.11 ± 0.01	0.13 ± 0.05	0.869	0.912	0.912	0.459
22:4n-6	0.1 ± 0.01 ^ab^	0.06 ± 0.02^ab^	0.11 ± 0.01 ^a^	0.05 ± 0.01 ^b^	0.025	0.809	0.006	0.482
22:5n-6	0.27 ± 0.06 ^a^	0.16 ± 0.03 ^b^	0.28 ± 0.02 ^a^	0.16 ± 0.02 ^b^	0.043	1.000	0.010	0.850
22:5n-3	2.72 ± 0.99	1.95 ± 0.3	3.21 ± 0.37	1.74 ± 0.31	0.170	0.747	0.050	0.442
22:6n-3	17.24 ± 7.69	17.76 ± 3.29	19.58 ± 3.12	15.75 ± 3.83	0.884	0.965	0.655	0.561
Total Saturates	25.35 ± 5.23	18.78 ± 0.83	22.62 ± 3.09	19.52 ± 0.42	0.266	0.671	0.090	0.468
Total Monoenes	31.87 ± 5.58	35.44 ± 1.85	30.48 ± 1.36	36.16 ± 2.39	0.357	0.892	0.114	0.669
Total n-3	32.32 ± 11.51	31.44 ± 3.28	37.39 ± 4.78	29.81 ± 3.51	0.714	0.734	0.421	0.517
Total n-6	8.66 ± 0.64 ^b^	13.33 ± 0.59 ^a^	7.61 ± 0.21 ^b^	13.53 ± 0.68 ^a^	0.001	0.343	<0.001	0.187
Total n-9	20.91 ± 3.75 ^ab^	28.62 ± 1.61 ^a^	18.84 ± 1 ^b^	29.26 ± 1.65 ^a^	0.021	0.677	0.005	0.442
Total n-3 LC-PUFA	29.67 ± 11.38	25.81 ± 4.1	34.39 ± 4.77	23.54 ± 4.36	0.493	0.814	0.204	0.511
EPA + DHA	25.76 ± 10.23	22.89 ± 3.75	29.88 ± 4.36	20.81 ± 4.07	0.556	0.828	0.246	0.519
ARA/EPA	0.13 ± 0.02	0.12 ± 0.00	0.09 ± 0.01	0.11 ± 0.01	0.168	0.056	0.621	0.345
EPA/ARA	8.15 ± 1.63	8.56 ± 0.16	11.01 ± 1.25	9.47 ± 0.09	0.157	0.061	0.480	0.253
DHA/ARA	16.37 ± 5.77	29.47 ± 3.39	20.91 ± 3.2	29.32 ± 5.99	0.120	0.551	0.033	0.525
DHA/EPA	1.98 ± 0.31 ^ab^	3.45 ± 0.33 ^a^	1.9 ± 0.08 ^b^	3.1 ± 0.6 ^ab^	0.033	0.463	0.008	0.647
n-3/n-6	3.79 ± 1.61	2.37 ± 0.35	4.93 ± 0.76	2.21 ± 0.37	0.114	0.497	0.034	0.378
18:4n-3/18:3n-3	0.58 ± 0.11 ^b^	0.11 ± 0.00 ^c^	0.85 ± 0.05 ^a^	0.1 ± 0.00 ^c^	0.001	0.043	<0.001	0.035
20:2n-9/20:1n-9	0.31 ± 0.07 ^b^	0.56 ± 0.03 ^a^	0.29 ± 0.01 ^b^	0.52 ± 0.03 ^a^	0.005	0.323	0.001	0.808
20:3n-6/20:2n-6	0.69 ± 0.1 ^ab^	0.51 ± 0.01 ^b^	0.82 ± 0.02 ^a^	0.54 ± 0.02 ^b^	0.012	0.102	0.003	0.265
20:4n-3/20:3n-3	5.03 ± 0.49 ^a^	1.96 ± 0.02 ^b^	6.62 ± 0.98 ^a^	1.9 ± 0.06 ^b^	0.002	0.119	0.001	0.101

Means bearing different superscript letters differ significantly (*p* < 0.05, One-way ANOVA, Tukey Post-Hoc).

## Data Availability

Data is contained within the article or Supplementary Materials. The data presented in this study are available in the article and Supplementary Materials.

## Supplementary Materials

The following are available online at https://www.mdpi.com/2076-2615/11/2/519/s1. Table S1: Pearson’s correlation coefficient of broodstock body weight, hepatosomatic, gonadosomatic index, steroid sex hormone levels of gilthead seabream male or female broodstock of high (HG) or low growth (LG) groups before feeding the experimental diets. Table S2: Regression relationship analysis between broodstock body weight and HSI, GSI, TST, 11KT and E2. Table S3: Pearson’s correlation coefficient of broodstock body weight, steroid sex hormone levels of gilthead seabream male or female broodstock of high (HG) or low growth (LG) groups fed with either FO or VO diet over three months of reproductive season. Table S4: Regression relationship analysis between broodstock body weight and steroid sex hormone levels of gilthead seabream male or female broodstock of high (HG) or low growth (LG) groups fed with either FO or VO diet over three months of reproductive season. Table S5: Pearson’s correlation coefficient of steroid sex hormone levels and sperm quality of gilthead seabream male broodstock of high (HG) or low growth (LG) groups fed with either FO or VO diet over three months of reproductive season. Table S6: Regression relationship analysis between steroid sex hormones and sperm quality of gilthead seabream male broodstock of high (HG) or low growth (LG) groups fed with either FO or VO diet over three months of reproductive season. Table S7: Pearson’s correlation coefficient of sperm quality and egg quality of gilthead seabream broodstock of high (HG) or low growth (LG) groups fed with either FO or VO diet over three months of reproductive season. Table S8: Regression relationship analysis between sperm quality and egg quality of gilthead seabream broodstock (combined data from high (HG) or low growth (LG) groups fed with either FO or VO diet over three months of reproductive season.

## Abbreviations

ALA	linolenic acid (18:3n-3)
ARA	arachidonic acid (20:4n-6)
BLUP	best linear unbiased prediction
EPA	eicosapentaenoic acid (20:5n- 3)
DHA	docosahexaenoic acid (22:6n-3)
fads2	fatty acyl desaturase (gene in fish)
Fads	fatty acyl desaturase (enzyme in fish)
FM	fish meal
FO	fish oil
LC-PUFA	long chain polyunsaturated fatty acid
HG	high growth
LA	linoleic acid (18:2n-6)
LG	low growth
