# Erratum: Ferosekhan et al. Influence of Genetic Selection for Growth and Broodstock Diet n-3 LC-PUFA Levels on Reproductive Performance of Gilthead Seabream, *Sparus Aurata.* *Animals* 2021, *11*, 519

**DOI:** 10.3390/ani11102957

**Published:** 2021-10-14

**Authors:** Shajahan Ferosekhan, Serhat Turkmen, Cathaysa Pérez-García, Hanlin Xu, Ana Gómez, Nazeemashahul Shamna, Juan Manuel Afonso, Grethe Rosenlund, Ramón Fontanillas, Anselmo Gracia, Marisol Izquierdo, Sadasivam Kaushik

**Affiliations:** 1 IU-ECOAQUA, Universidad de Las Palmas de Gran Canaria, Taliarte, 35214 Telde, Spain; turkmen@uab.edu (S.T.); cathaysa.perez108@alu.ulpgc.es (C.P.-G.); hanlinxuulpgc@outlook.com (H.X.); juanmanuel.afonso@ulpgc.es (J.M.A.); anselmo.gracia@ulpgc.es (A.G.); marisol.izquierdo@ulpgc.es (M.I.); sachi.kaushik@ulpgc.es (S.K.); 2 ICAR-Central Institute of Freshwater Aquaculture, Bhubaneswar 751002, Odisha, India; 3 Department of Biology, University of Alabama at Birmingham, Birmingham, AL 35294, USA; 4 Institute of Aquaculture Torre de la Sal (IATS), CSIC, Ribera de Cabanes, 12595 Castellόn, Spain; a.gomez@csic.es; 5 ICAR-Central Institute of Fisheries Education, Mumbai 400061, Maharashtra, India; shamna@cife.edu.in; 6 Skretting Aquaculture Research Centre, Sjohagen, 4016 Stavanger, Norway; Grethe.Rosenlund@skretting.com (G.R.); ramonfontanillas@gmail.com (R.F.)

The authors wish to make the following corrections to this paper [1].

1. Delete “Acknowledgments”.
2. Change in Funding.
