# Supplementary material for: Influence of Genetic Selection for Growth and Broodstock Diet n-3 LC-PUFA Levels on Reproductive Performance of Gilthead Seabream, Sparus aurata
Source: Animals (Basel). 2021 Feb 17;11(2):519. doi: 10.3390/ani11020519 (PMC7922623; doi:10.3390/ani11020519)
Supplement: Supplementary file 1 [file animals-11-00519-s001.pdf]

Supplementary tables

INFLUENCE OF GENETIC SELECTION FOR GROWTH AND BROODSTOCK DIET n-3 LC-PUFA LEVELS ON REPRODUCTIVE PERFORMANCE OF GILTHEAD SEABREAM, *Sparus aurata*

Shajahan Ferosekhan, Serhat Turkmen, Cathaysa Pérez-García, Hanlin Xu, Ana Gómez, Nazeemashahul Shamna, Juan Manuel Afonso, Grethe Rosenlund, Ramón Fontanillas, Anselmo Gracia, Marisol Izquierdo and Sadasivam Kaushik

Table S1. Pearson's correlation coefficient of broodstock body weight, hepatosomatic, gonadosomatic index, steroid sex hormone levels of gilthead seabream male or female broodstock of high (HG) or low growth (LG) groups before feeding the experimental diets

Pearson's correlation	HSI %	GSI %	TST ng/ml	11KT ng/ml	E2 ng/ml
Broodstock body weight (g)	−0.15	0.26	0.22	−0.01	0.06
HSI %	-	−0.22	−0.511 *	−0.466 *	−0.04
GSI %	−0.22	-	−0.17	−0.22	0.01
TST (ng/ml)	−0.511 *	−0.17	-	0.852 **	0.500 *
11KT (ng/ml)	−0.466 *	−0.22	0.852 **	-	0.24
E2 (ng/ml)	−0.04	0.01	0.500 *	0.24	-

The symbol *, ** denotes significant differences as $P < 0.05$ or 0.01 level

Table S2. Regression relationship analysis between broodstock body weight and HSI, GSI, TST, 11KT and E2

**Regression analysis**	**HSI %**		**GSI %**		**TST ng/ml**		**11KT ng/ml**		**E2 ng/ml**	
	**R^2**	***P***	**R^2**	***P***	**R^2**	***P***	**R^2**	***P***	**R^2**	***P***
Broodstock body weight	0.034	0.422	0.083	0.204	0.049	0.335	0.015	0.949	0.004	0.796

Table S3. Pearson's correlation coefficient of broodstock body weight, steroid sex hormone levels of gilthead seabream male or female broodstock of high (HG) or low growth (LG) groups fed with either FO or VO diet over three months of reproductive season

**Male broodstock**	**Body weight**	**Testosterone (ng/ml)**	**11 Keto-testosterone (ng/ml)**
Body weight	-	−0.29	−0.504 *
Testosterone (ng/ml)	−0.29	-	0.699 **
11 Keto-testosterone (ng/ml)	−0.504 *	0.699 **	-
**Female broodstock**	**Body weight**	**Testosterone (ng/ml)**	**17β-estradiol (ng/ml)**
Body weight	-	−0.098	−0.052
Testosterone (ng/ml)	−0.098	-	−0.134
17β-estradiol (ng/ml)	−0.052	−0.134	-

The symbol *, ** denotes significant differences as $P < 0.05$ or 0.01 level

Table S4. Regression relationship analysis between broodstock body weight and steroid sex hormone levels of gilthead seabream male or female broodstock of high (HG) or low growth (LG) groups fed with either FO or VO diet over three months of reproductive season

**Regression analysis**	**Testosterone (ng/ml)**		**11 Keto-testosterone (ng/ml)**		**17β-estradiol (ng/ml)**	
	**R^2**	***P***	**R^2**	***P***	**R^2**	***P***
Male body weight	0.085	0.257	0.254	**0.039**	-	-
Female body weight	0.010	0.707	-	-	0.004	0.816

Table S5. Pearson's correlation coefficient of steroid sex hormone levels and sperm quality of gilthead seabream male broodstock of high (HG) or low growth (LG) groups fed with either FO or VO diet over three months of reproductive season

**Pearson's correlation**		**Sperm concentration (10^9 cells / ml)**	**Sperm viability %**	**Sperm motility %**	**Sperm motility duration (sec)**
Testosterone (ng/ml)	r	−0.456	0.100	−0.250	−0.086
	P	0.117	0.744	0.411	0.780
11 Keto-testosterone (ng/ml)	r	−0.544	−0.162	−0.510	−0.259
	P	0.055	0.598	0.075	0.394
Sperm concentration (10^9 cells / ml)	r	-	0.053	0.635*	0.057
	P	-	0.858	0.015	0.848
Sperm viability %	r	0.053	-	0.411	0.521
	P	0.858	-	0.144	0.056
Sperm motility %	r	0.635*	0.411	-	0.450
	P	0.015	0.144	-	0.106
Sperm motility duration (sec)	r	0.057	0.521	0.450	-
	P	0.848	0.056	0.106	-

The symbol *, ** denotes significant differences as $P < 0.05$ or 0.01 level

Table S6. Regression relationship analysis between steroid sex hormones and sperm quality of gilthead seabream male broodstock of high (HG) or low growth (LG) groups fed with either FO or VO diet over three months of reproductive season

**Regression analysis**	**Sperm Concentration (10^9 cells / ml)**		**Sperm viability %**		**Sperm motility %**		**Sperm motility duration (sec)**	
	**R^2**	***P***	**R^2**	***P***	**R^2**	***P***	**R^2**	***P***
Testosterone (ng/ml)	0.305	0.050	0.010	0.744	0.062	0.411	0.07	0.780
11 Keto-testosterone (ng/ml)	0.409	0.018	0.026	0.598	0.261	0.075	0.067	0.394

Table S7. Pearson's correlation coefficient of sperm quality and egg quality of gilthead seabream broodstock of high (HG) or low growth (LG) groups fed with either FO or VO diet over three months of reproductive season

Pearson's correlation		Sperm concentration (10^9 cells/ml)	Sperm viability %	Sperm motility %	Sperm motility duration (sec)	Egg fertilization %	Egg viability %	Hatching %	Larval survival (3dph) %
Sperm concentration (10^9 cells/ml)	r	-	−0.268	0.884	−0.090	0.289	0.245	−0.380	−0.202
	P	-	0.732	0.116	0.910	0.711	0.755	0.620	0.798
Sperm viability %	r	−0.268	-	0.094	0.908	0.997 **	0.957*	−0.710	−0.874
	P	0.732	-	0.906	0.092	0.003	0.043	0.290	0.126
Sperm motility %	r	0.884	0.094	-	0.358	−0.094	−0.009	−0.506	−0.453
	P	0.116	0.906	-	0.642	0.906	0.991	0.494	0.547
Sperm motility duration (sec)	r	−0.090	0.908	0.358	-	−0.929	−0.758	−0.592	−0.796
	P	0.910	0.092	0.642	-	0.071	0.242	0.408	0.204
Egg fertilization %	r	0.289	0.997**	−0.094	−0.929	-	0.934	0.669	0.849
	P	0.711	0.003	0.906	0.071	-	0.066	0.331	0.151
Egg viability %	r	0.245	0.957*	−0.009	−0.758	0.934	-	0.797	0.893
	P	0.755	0.043	0.991	0.242	0.066	-	0.203	0.107
Hatching %	r	−0.380	−0.710	−0.506	−0.592	0.669	0.797	-	0.957 *
	P	0.620	0.290	0.494	0.408	0.331	0.203	-	0.043
Larval survival (3dph) %	r	−0.202	−0.874	−0.453	-0.796	0.849	0.893	0.957 *	-
	P	0.798	0.126	0.547	0.204	0.151	0.107	0.043	-

The symbol *, ** denotes significant differences as $P < 0.05$ or 0.01 level

Table S8. Regression relationship analysis between sperm quality and egg quality of gilthead seabream broodstock (combined data from high (HG) or low growth (LG) groups fed with either FO or VO diet over three months of reproductive season

**Regression analysis**	**Egg fertilization %**		**Egg viability %**		**Hatching %**		**Larval survival (3dph) %**	
	**R^2**	**P**	**R^2**	**P**	**R^2**	**P**	**R^2**	**P**
Sperm concentration (10^9 cells/ml)	0.083	0.711	0.060	0.755	0.145	0.620	0.041	0.798
Sperm viability %	0.995	0.003	0.915	0.043	0.505	0.290	0.763	0.126
Sperm motility %	0.009	0.906	0.001	0.991	0.256	0.494	0.205	0.547
Sperm motility duration (sec)	0.863	0.071	0.574	0.242	0.350	0.408	0.633	0.204
